# Increased prevalence and incidence of anemia among adults in transforming rural China: two cross-sectional surveys

**DOI:** 10.1186/s12889-015-2671-8

**Published:** 2015-12-28

**Authors:** Xuecai Wang, Zhaofan Wu, Yue Chen, Jianfu Zhu, Xiaolian Dong, Chaowei Fu, Qingwu Jiang

**Affiliations:** Deqing County Center of Disease Prevention and Control, Deqing, Zhejiang Province China; Department of Epidemiology, School of Public Health and Key Laboratory of Public Health Safety, Fudan University, 138 Yi Xue Yuan Road, Shanghai, 200032 China; Faculty of Medicine, University of Ottawa, Ottawa, Ontario Canada

**Keywords:** Anemia, Prevalence, Incidence, Rural China

## Abstract

**Background:**

Anemia remains one of the serious nutrition-related diseases in China, but data on incidence of anemia were less available, especially in rural area which are experiencing rapid urbanization. Out study aimed to estimate both the prevalence and incidence of anemia in transforming rural China.

**Methods:**

We conducted a combined study of rural adults 18–64 years of age with a repeated cross-sectional component (4456 in 2006 and 2184 in 2008) and a cohort component (1424) in rural Deqing, China. Anemia was diagnosed based on blood hemoglobin levels using the hemiglobincyanide (HiCN) method according to both the World Health Organization (WHO) and Chinese criteria. The prevalence and incidence of anemia and their 95 % confidential intervals (95 % CI) were calculated.

**Results:**

The prevalence of anemia based on the WHO criteria was 51.5 % in 2006 and 53.7 % in 2008, and the 2-year cumulative incidence was 42.1 %. Of the cases, over 95 % had mild anemia. The prevalence was much lower when the Chinese criteria was used. Both the prevalence and incidence were higher in women than in men and significantly increased with age in men. In both sexes, the incidence sharply increased after 45 years of age.

**Conclusion:**

Our study showed a high prevalence and incidence of anemia among adults in rural Deqing, China. Monitoring and intervention were needed urgently, especially among individuals over 45 years of age.

**Electronic supplementary material:**

The online version of this article (doi:10.1186/s12889-015-2671-8) contains supplementary material, which is available to authorized users.

## Background

 Anemia is one of the serious nutrition-related diseases, which is associated with increased morbidity and mortality, and can lead to cardiovascular and neurological events [1, 2]. The World Health Organization (WHO) estimated that 1.62 billion people worldwide were affected by anemia in the period of 1993–2005, which corresponds to 24.8 % of the population, and most of them were in developing countries including China [3, 4]. A nationwide survey conducted in China indicated that 20.1 % of residents were affected by anemia in 2002 [5, 6].

Since early 1980s, China had been experiencing great changes in nutrition, health care and aging, which were associated with the occurrence of anemia; however, few surveys studied the changes in the prevalence and incidence of anemia [7–9]^.^, especially in transforming rural China [10–12] . One difficulty was that there were two different diagnostic criteria for anemia used in China: WHO and Chinese (CN) criteria. The WHO criteria [3] was generally accepted for public health research and CN one was commonly used in clinical diagnosis [13] . The difference in the estimation of prevalence and incidence between the two criteria in Chinese population and to what extent had rarely been studied so far.

Deqing County is one of the most developed areas in China [14], and has been experiencing rapid urbanization. This study was carried out to estimate the prevalence and its change over a 2-year period, and the incidence of anemia. We also compared the estimates for anemia occurrence using the WHO and Chinese criteria.

## Methods

### Study population

**Fig. 1:**
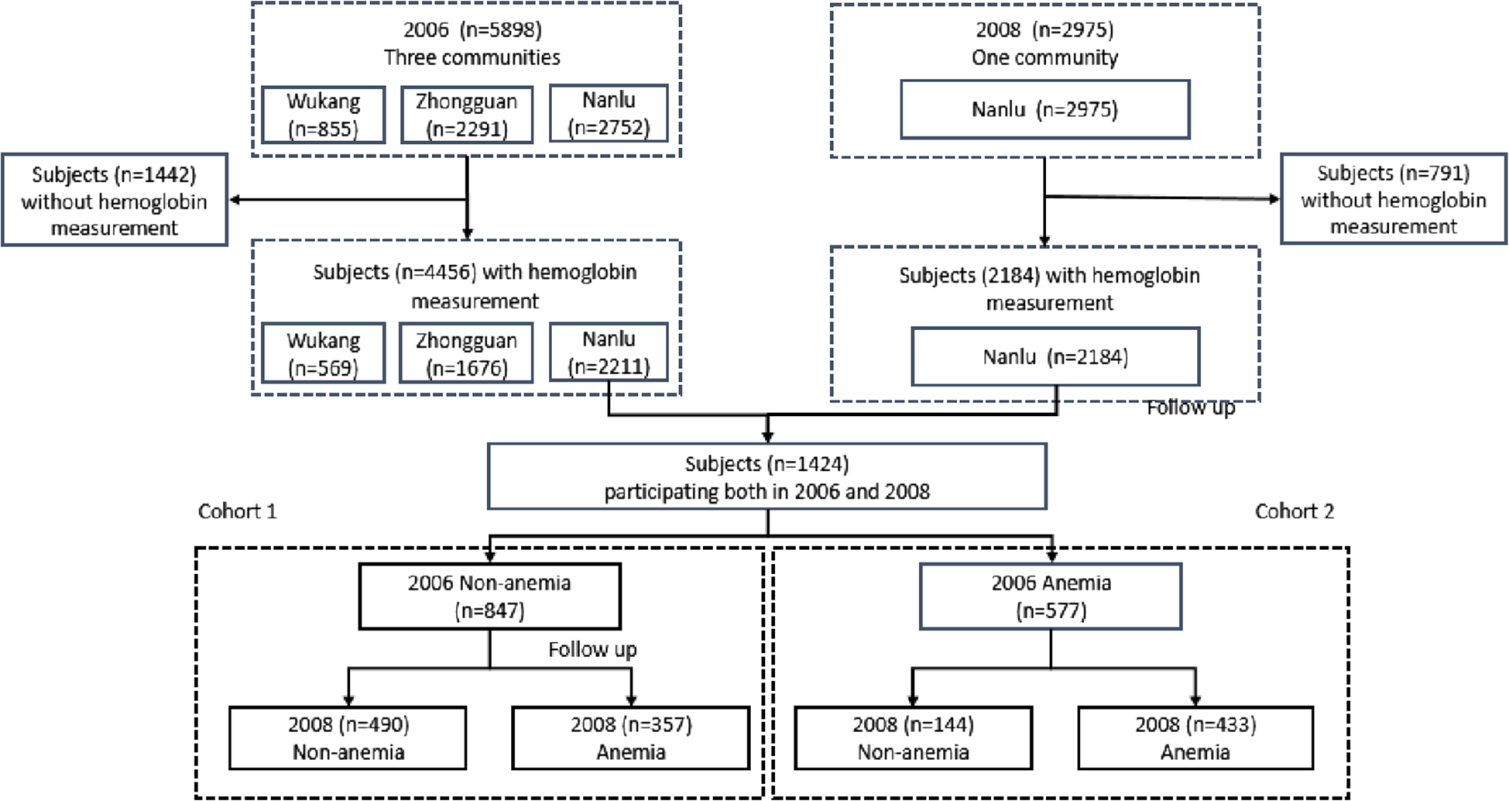
The work flowchart of the study design

A combined study with a repeated cross-sectional component and a cohort component was conducted in rural Deqing County, Zhejiang Province, China. A total of 5898 participants were randomly cluster-sampled from three rural communities (Nanlu, Wukang and Zhongguan) in 2006 [15] and 4456 (76 %) of them had hemoglobin measurement. A repeated cross-sectional study was carried out in the largest community (Nanlu) of these three rural communities in 2008 and 2975 subjects were recruited with 2184 subjects having hemoglobin measurement. Of 2211 subjects who participated the 2006 survery in Nanlu Community, 1424 (577 had anemia and 847 had no anemia in 2006) were followed up in 2008 (Fig. 1).

Inclusion criteria of subjects were those who 1) were aged 18–64 years old; 2) were local residents living for more than 6 months during the past 12 months in those rural communities; 3) provided written informed consent; and 4) answered the questions independently. Temporary workers or students living in the communities for no more than 6 months during the past 12 months were excluded from the study.

### Data collection

Local health workers who were recruited and trained by academic investigators, conducted face-to-face interviews. A questionnaire covered the information on demographic factirs (including birthday, gender, marriage, education years, occupation, family size, family incoming, and so on) and behavioral factors (including smoking, alcohol drinking, tea drinking, diet and physical exercise). For those who had fasted for more than 8 h, physical examinations were carried out including height, weight, wais circumference, heart rate, blood pressure, electrocardiogram and type-B ultrasonic. Venous blood of 5 ml were collected and preserved under −20 °C with and without anticoagulant. Hemoglobin was measured within 24 h after collecting blood using the hemiglobincyanide (HiCN) method.

### Definition of anemia

Anemia was diagnosed based on blood hemoglobin levels using both the WHO criteria (hemoglobin <130 g/L for men or <120 g/L for women and 90 g/L < hemoglobin <130 g/L for men or 90 g/L < hemoglobin < 120 g/L for women as mild anemia) and the Chinese criteria (hemoglobin <120 g/L for men or <110 g/L for women) [3, 16].

### Data analysis

All statistical tests were performed using SPSS17.0 (SPSS Inc, Chicago, Illinois, USA). For comparison, Chi-square test was used for categorical variables, and independent *t* test for continuous variables. The prevalence and cumulative incidence of anemia and their 95 % confidential intervals (95 % CI) were calculated. An alpha level of <0.05 (two sides) was considered to be statistically significant.

## Results

The 2006 survey included 4456 participants with hemoglobin measured and the 2008 survey included 2184. Participants in the two cross-sectional studies had similar average age and BMI and similar distributions of gender, education level and physical exercise (*p >* 0.05). However, the study population had higher proportions of low-income families, famers, smokers, drinkers and mainly vegetable eaters for the 2008 survey compared with the 2006 survey (*p <* 0.001) (Table 1).

**Table 1:** Characteristics of participants in 2006 and 2008

	2006 (*n* = 4456)	2008 (*n* = 2184)	
	Mean ± Deviation	Mean ± Deviation	*χ*2, *p* value
Age (years)	46.8 ± 9.5	46.8 ± 9.4	0.1, 0.952
BMI (kg/m2)	22.1 ± 3.7	22.1 ± 3.9	0.2, 0.847
	No.[%]	No.[%]	t, *p* value
Gender			1.8, 0.175
Men	1846 [41.4]	943 [43.2]	
Women	2610 [58.6]	1241 [56.8]	
Education			3.6, 0.057
Middle school or above	1703 [38.4]	887 [40.8]	
Below middle school	2731 [61.6]	1285 [59.2]	
Household income level			49.5, <0.001
Low	492 [11.1]	377 [17.3]	
Medium	3314 [74.6]	1514 [69.4]	
High	634 [14.3]	289 [13.3]	
Occupation			53.3, <0.001
Famer	3206 [72.4]	1753 [80.7]	
Non-famer	1222 [27.6]	420 [19.3]	
Smoking			11.7, <0.001
Yes	1227 [27.6]	690 [31.7]	
NO	3216 [72.4]	1489 [68.3]	
Alcohol use			17.3, <0.001
Yes	938 [21.1]	559 [25.6]	
NO	3518 [78.9]	1625 [74.4]	
Physical exercise			2.4, 0.125
Yes	93 [2.2]	59 [2.8]	
NO	4125 [97.8]	2022 [97.2]	
Dietary chief component			18.7, <0.001
Vegetable	622 [14.0]	391 [18.0]	
Meat	314 [7.1]	160 [7.4]	
Vegetable and Meat	3493 [78.9]	1617 [74.6]	

Mean hemoglobin levels were 123.5 ± 19.3 g/L in 2006 similar to that of 123.3 ± 16.7 g/L in 2008 (t = 0.49, *P* = 0.626). According to the WHO criteria, the prevalence of anemia was 51.5 % (95 % CI: 50.0–52.9 %) in 2006 and 53.7 % (95 % CI: 51.6–55.8 %) in 2008. Over 95 % of the patients were considered to have mild anemia. In the follow-up study of 847 participants without anemia in 2006, the 2-year cumulative incidence of anemia was 42.1 % (95 % CI: 38.8–45.5 %). Also, 577 subjects with anemia in 2006 were followed up, and 24.9 % (95 % CI: 21.4–28.5 %) of them recovered from anemia in 2008. When the Chinese criteria was used, the prevalence and 2-year cumulative incidence of anemia were 24.5 and 22.1 %, respectively (Tables 2 and 3).

**Table 2:** Prevalence of anemia (%) according to WHO and Chinese criteria among adults in rural Deqing

WHO criteria						
Age (years)	2006 (*n* = 4456)			2008 (*n* = 2184)		
	Men	Women	Total	Men	Women	Total
18–34^a^	38(20.5)	196(63.4)	234(47.4)	12(19.4)	79(73.8)	91(53.8)
35–44	169(29.5)	571(64.7)	740(50.8)	64(25.7)	232(64.8)	296(48.8)
45–54	232(35.9)	525(61.7)	757(50.5)	155(40.8)	287(64.8)	442(53.7)
55–64	199(45.1)	364(64.2)	563(55.9)	120(45.5)	218(70.6)	338(59.1)
Total	638(34.6)	1656(63.4)	2294(51.5)	351(36.8)	816(67.1)	1167(53.7)
*χ*2, p	44.8,<0.001	1.8, 0.67	11.8, 0.008	36.1,<0.001	7.2, 0.67	14.1, 0.003
*χ*2 _trend_, p	45.6,<0.001	1.83, 0.61	8.9, 0.003	37.9,<0.001	7.3, 0.62	14.2, 0.003
Chinese criteria						
18–34^a^	9(4.9)	108(35.0)	117(23.7)	4(6.5)	47(43.9)	51(30.2)
35–44	53(9.2)	321(36.4)	374(25.7)	18(7.2)	116(32.4)	134(22.1)
45–54	77(11.9)	281(33.0)	358(23.9)	62(16.3)	132(29.8)	194(23.6)
55–64	96(21.8)	214(37.7)	310(30.8)	46(17.4)	107(34.6)	153(26.7)
Total	235(12.7)	924(35.4)	1159(26.0)	130(13.6)	402(33.1)	532(24.5)
*χ*2, p	49.4,<0.001	3.8, 0.28	16.7, 0.001	17.5, 0.001	12.0, 0.007	9.0, 0.300
*χ*2 _trend_, p	47.9,<0.001	3.9, 0.28	16.4, 0.001	19.4,<0.001	11.6, 0.009	8.7, 0.033

^a^18–24 and 25–34 year age groups were combined due to small sizes

**Table 3:** Cumulative incidence of anemia (%) according to WHO and Chinese criteria among adults in rural Deqing

Age(years)	Men		Women		Total	
	WHO	CN	WHO	CN	WHO	CN
18–34	4(13.3)	3(8.3)	27(67.5)	21(35.1)	31(44.3)	24(25.1)
35–44	32(23.2)	21(12.4)	67(50.0)	51(23.4)	99(36.4)	72(18.6)
45–54	54(32.3)	22(10.5)	84(56.0)	56(25.7)	138(43.5)	78(18.2)
55–64	35(36.8)	19(16.2)	54(58.1)	36(27.3)	89(47.3)	55(22.1)
Total	125(29.1)	65(12.2)	232(55.6)	164(26.1)	357(42.1)	229(19.7)
*χ*2, p	9.6, 0.023	2.9, 0.412	4.2, 0.247	3.4, 0.334	6.1, 0.105	3.5, 0.324
χ2_trend_, p	10.1, 0.018	2.8, 0.422	4.3, 0.232	3.3, 0.351	6.2, 0.103	3.4, 0.338

*CN* Chinese criteria

The prevalence and 2-year cumulative incidence of anemia were both significantly higher among women than men in all age groups, and the difference gradually narrowed as age rose. The 2-year cumulative incidence increased significantly with age in men, while it was the highest in the 18–34-year age group, then dropped slightly in the 35–44-year age group and gradually increased again after 45 years of age in women. In both sexes, the incidence sharply increased after 45 years of age (Tables 2 and 3).

## Discussion

In the current study, about half of the study population had anemia in Deqing, which was far higher than the estimates from previous studies, such as 16.2 % in rural China in 2002 [17], 18.9 % in Zhejiang province, China in 2005 [18], 17.1 % in Japan [19] and 24.8 % for the global population [4] . Some limited epidemiologic data on the incidence of anemia among general populations [8] revealed that the incidence in elderly was 24.0 per 1000 person-years in Korea [9], 24.2 per 1000 person-years in Italy [8] and 5.8 per 1000 person-years in Germany [7]. Compared with these results, the incidence (approximately 220 per 1000 person-years) in the present study was extremely high. Several reasons may explaine the high incidence. 1) Data from the Fourth China Health and Nutrition Survey demonstrated that hemoglobin levels among Chinese were lower than those from other countries in general [13]. 2) In rural Deqing, meat that contains high iron accounts for small proportions of local diet. We found that subjects who had vegetable and meat evenly, had a lower risk of anemia than those having vegetable mainly, after adjustment for age, gender, education, income and BMI. 3) In the current study, although about a half of subjects were anemic, most of them had mild anemia which is considered to have a minimal effect on health. One-fourth of patients recovered form anemia without any treatment over a 2-year period.

Rural areas of China have been undergoing a rapid urbanization during the past several decades. In one way, as family income increases, there is an changing pattern of diet. It is obaserved that more energy rather than nutrition has been taken among some Chinese residents [20], which may not help in improving their nutrition status overall. In another way, a larger proportion of participants, especially older people and women, mainly consumed vegetables. This may be one reason for the observation that more than 40 % develped anemia but only one quarter recovered from anemia during two-year period in this study.

The Fourth China Health and Nutrition Survey showed that the quartile range of hemoglobin in Chinese was lower than those from other countries [13]. Because the cut-off points for anemia for the CN criteria, regardless of gender, were 10 g/L lower than those for the WHO criteria (WHO vs CN: 130 vs 120 g/L for men; 120 vs 110 g/L for women) [3, 16]. The 10 g/L difference between the two criteria has made huge differences in the estimation of both prevalence and incidence since about half of patients with anemia based on the WHO criteria in rural Deqing had their hemoglobin levels in 120–130 g/L among men and 110–120 g/L among women. Anemia is considered to be one of the most serious nutrition-related diseases in China, and more researches should be done to explore health hazards with different criteria of anemia to develop proper cut-off points of anemia for Chinese populaiton.

Our results revealed higher prevalence and incidence of anemia among women than men in all age groups, and the sex differences gradually narrowed as age rose. Iron deficiency can be caused by menstruation, pregnancy and lactation in women, and the impact on the sex difference may diminich with age, especially after menopause [21, 22]. In another aspect, however, the prevalence and incidence anemia increased with age, especially in men, which was consistent with results from a system review [23] and an age dependency of anemia occurrence was also found in a longitudinal Swedish study [24] . For both sex, we observed a relatively higher prevalence and incidence among those 45 years of age or older, and previous studies from Korea and the United State used age cutpoint of 49 years and found similar results [25, 26]. Older adults were found to have lower levels of hemoglobin and hematocrit than younger adults because of decreasing physiological functions and studies suggested to use the hemoglobin level of <110 g/L as the diagnostic criteria for older adults [27, 28]. Our finding indicated that the age of 45 years may be an important age point for the prevention of anemia for rural Chinese,

The findings of our study should be considered with awareness of its limitations. First, only a portion (64.4 %) of the subjects were followed up, who were slightly older, more likely to be woman, having lower education than those who were not followed up (Additional file 1: Table S1), which might result in an overestimated incidence. Secondly, although all blood were tested with the same method and equipment at the Deqing Center for Disease Control, measurement errors might exit and vary. However, we have no reason to believe that there exit a systematic error for the observed high prevalence and incidence in the context that such equipment was evaluated and adjusted every year in a national center for laboratory equipment and testing, which was done under a standard operation procedure. Thirdly, the study population was from more developed rural area of China, and therefore extra caution should be taken when the findings from this study are generalized to more broader populations with different socioeconomic status. In addition, there may be some impacts from unmeasured factors such as vitamin B12 and genetic variants, and for repeated surveys, part of the anemia variation might be due to the regression towards mean.

## Conclusion

In conclusion, anemia was prevalent among the community population aged 18–64 years in rural Deqing, which calls for interventions, especially for those aged 45 years old or above. The WHO and Chinese criteria for anemia showed marked differences in the estimation of prevalence and incidence for this rural population. More researches are needed to explore health hazards associated with different criteria of anemia and to develop proper cut-off points of hemoglobin for anemia diagnosis in Chinese populaiton.

### Ethics approval

The study was approved by the Institutional Review Board of the Fudan University School of Public Health. A written informed consent was obtained after a complete description of the study narrated to each subject.

## Additional file

Additional file 1: Table S1: Comparison of characteristics between participants followed up and those not. (DOC 46 kb)

## Abbreviations

HiCN: hemiglobincyanide
WHO: World Health Organization
CN: Chinese criteria
BMI: Body Mass Index
CDC: Center for Disease Control
