# Supplementary material for: Increased prevalence and incidence of anemia among adults in transforming rural China: two cross-sectional surveys
Source: BMC Public Health. 2015 Dec 28;15:1302. doi: 10.1186/s12889-015-2671-8 (PMC4693413; doi:10.1186/s12889-015-2671-8)
Supplement: Additional file 1: Table S1: — Comparison of characteristics between participants followed up and those not. (DOC 46 kb) [file 12889_2015_2671_MOESM1_ESM.doc]

**Additional file 1: Table S1** Comparison of characteristics between participants followed up and those not

| Variable | Follow-up (n=1424) | Non-follow-up (n=787 ) |  |
| --- | --- | --- | --- |
|  | Mean ± Deviation | Mean ± Deviation | χ2, p value |
| Age (years) | 47.5 ± 8.8 | 45.2 ± 10.3 | 5.4, <0.001 |
| BMI (kg/m2) | 22.6 ± 2.9 | 22.1 ± 2.7 | 4.5, <0.001 |
| Baseline Hemoglobin(g/L) | 126.6 ± 16.8 | 127.3 ± 17.1 | 0.8, 0.403 |
|  | NO. [%] | NO. [%] | t, p value |
| Gender |  |  | 6.2, 0.013 |
| Men | 586 [41.2] | 367 [46.6] |  |
| Women | 838 [58.8] | 420 [53.4] |  |
| Education |  |  | 9.9, 0.002 |
| Middle school or above | 546 [38.6] | 356 [45.5] |  |
| Below middle school | 870 [61.4] | 427 [54.5] |  |
| Household income |  |  | 14.7, <0.001 |
| Low | 234 [16.4] | 148 [18.9] |  |
| Medium | 971 [68.2] | 559 [71.4] |  |
| High | 219 [15.4] | 76 [9.7] |  |
| Occupation |  |  | 4.7, 0.030 |
| Famer | 1161 [81.8] | 609 [78.0] |  |
| Non-famer | 258 [18.2] | 172 [22.0] |  |
| Smoking |  |  | 5, 0.026 |
| Yes | 425 [29.9] | 271 [34.5] |  |
| NO | 996 [70.1] | 514 [65.5] |  |
| Alcohol use |  |  | 01, 0.910 |
| Yes | 365 [25.6] | 200 [25.4] |  |
| NO | 1059 [74.4] | 587 [74.6] |  |
| Physical exercise |  |  | 1.6, 0.204 |
| Yes | 34 [2.5] | 26 [3.5] |  |
| NO | 1323 [97.5] | 724 [96.5] |  |
| Dietary chief component | |  | 0.7, 0.707 |
| Vegetable | 252 [17.8] | 144 [18.4] |  |
| Meat | 99 [7.0] | 61 [7.8] |  |
| Vegetable and Meat | 1063 [75.2] | 576 [73.8] |  |
